# Evaluation of Resting Spatio-Temporal Dynamics of a Neural Mass Model Using Resting fMRI Connectivity and EEG Microstates

**DOI:** 10.3389/fncom.2019.00091

**Published:** 2020-01-17

**Authors:** Hidenori Endo, Nobuo Hiroe, Okito Yamashita

**Affiliations:** ^1^Graduate School of Information Science, Nara Institute of Science and Technology, Nara, Japan; ^2^ATR Neural Information Analysis Laboratories, Kyoto, Japan; ^3^Center for Advanced Intelligence Project, RIKEN, Tokyo, Japan

**Keywords:** resting-state networks, resting-state functional connectivity, microstates, neural mass model, cortico-cortical dynamics

## Abstract

Resting-state brain activities have been extensively investigated to understand the macro-scale network architecture of the human brain using non-invasive imaging methods such as fMRI, EEG, and MEG. Previous studies revealed a mechanistic origin of resting-state networks (RSNs) using the connectome dynamics modeling approach, where the neural mass dynamics model constrained by the structural connectivity is simulated to replicate the resting-state networks measured with fMRI and/or fast synchronization transitions with EEG/MEG. However, there is still little understanding of the relationship between the slow fluctuations measured with fMRI and the fast synchronization transitions with EEG/MEG. In this study, as a first step toward evaluating experimental evidence of resting state activity at two different time scales but in a unified way, we investigate connectome dynamics models that simultaneously explain resting-state functional connectivity (rsFC) and EEG microstates. Here, we introduce empirical rsFC and microstates as evaluation criteria of simulated neuronal dynamics obtained by the Larter-Breakspear model in one cortical region connected with those in other cortical regions based on structural connectivity. We optimized the global coupling strength and the local gain parameter (variance of the excitatory and inhibitory threshold) of the simulated neuronal dynamics by fitting both rsFC and microstate spatial patterns to those of experimental ones. As a result, we found that simulated neuronal dynamics in a narrow optimal parameter range simultaneously reproduced empirical rsFC and microstates. Two parameter groups had different inter-regional interdependence. One type of dynamics was synchronized across the whole brain region, and the other type was synchronized between brain regions with strong structural connectivity. In other words, both fast synchronization transitions and slow BOLD fluctuation changed based on structural connectivity in the two parameter groups. Empirical microstates were similar to simulated microstates in the two parameter groups. Thus, fast synchronization transitions correlated with slow BOLD fluctuation based on structural connectivity yielded characteristics of microstates. Our results demonstrate that a bottom-up approach, which extends the single neuronal dynamics model based on empirical observations into a neural mass dynamics model and integrates structural connectivity, effectively reveals both macroscopic fast, and slow resting-state network dynamics.

## Introduction

Research on resting-state networks is attracting much attention in human neuroimaging. Resting-state functional connectivity (rsFC), i.e., co-activation patterns of slowly fluctuating BOLD signals measured with fMRI (on the order of seconds), has shown interesting empirical evidence on functional subnetworks and their relevance to individual differences (Smith et al., 2013). On the other hand, the microstates, i.e., fast-transient spatial patterns of human scalp potential measured with EEG (on the order of 10–100 ms), have been regarded as the building blocks of human information processing, and four canonical microstates appear in resting-state consistently across subjects and studies (Pascual-Marqui et al., 1995; Koenig et al., 2002; Michel and Koenig, 2018a). In addition, simultaneous fMRI and EEG measurements have been used to reveal the relationship between the slow fluctuation related to rsFC and the fast synchronization transition related to microstates in terms of the spatiotemporal dynamics of the human brain's information processing (Britz et al., 2010; Van de Ville et al., 2010; Yuan et al., 2012; Schwab et al., 2015; Bréchet et al., 2019). However, few mechanistic explanations of these two phenomena have been presented.

Recently, the connectome dynamics models, based on models of neural dynamics constrained by the whole brain's structural connectivity (called connectome), have been investigated to clarify the generative mechanism of functional brain activities and networks. Several computational studies have used simulated neuronal dynamics to understand the mechanistic origins of rsFC patterns (Breakspear et al., 2007; Honey et al., 2009; Deco and Jirsa, 2012; Deco et al., 2013), dynamic rsFC patterns (Hansen et al., 2015; Fukushima and Sporns, 2018), and static FC related to fast synchronization measured by MEG (Nakagawa et al., 2014; Deco et al., 2017; Abeysuriya et al., 2018). Furthermore, recent studies have tried to uncover the relationships between fast synchronization transition and slow fluctuation by combining experimental fMRI with EEG or/and MEG data. Schirner et al. proposed a connectome dynamics model that has EEG source currents in the alpha band as input and demonstrated that the model replicated multiple experimental observations measured with fMRI (Schirner et al., 2018). Demirtaş et al. proposed a locally heterogeneous connectome dynamics model that improved the replication performance of rsFC and MEG power spectrum spatial distribution (Demirtaş et al., 2019). Roberts et al. showed that the Larter-Breakspear model (Sanz-Leon et al., 2015) constrained by the connectome generated rich repertoires of rapidly changing spatiotemporal patterns that are in agreement with the temporal statistics of experimental data such as electrical waves in cortical tissue, sequential spatiotemporal patterns in the resting state MEG data, and large-scale waves in human electrocorticography as well as static rsFC (Roberts et al., 2019). However, similarities between experimental and simulated fast-transient spatial patterns have not yet been investigated.

In this study, to evaluate experimental evidence of resting-state activity on two different time scales but in a unified way, we investigated a connectome dynamics model that explains both experimental rsFC and microstates. We used the Larter-Breakspear model, in which the inhibitory and excitatory neurons in one region are connected with those in other regions based on a connectome measured with diffusion MRI. We optimized the global coupling strength and the local gain parameter (variance of the excitatory and inhibitory threshold) of the simulated neuronal dynamics by fitting both rsFC and microstate spatial patterns to those of the experimental ones. As a result, we found that fast synchronization transitions correlated with slow BOLD fluctuation based on structural connectivity yielded characteristics of empirical microstates. In detail, we found that the parameter sets with high fitting performance to rsFC overlapped with those with high fitting performance to microstates and that the optimal parameter range was greatly reduced by adding microstates as evaluation criteria compared with not adding them as in a previous work (Honey et al., 2009). We found two parameter regions where both rsFC and microstate spatial patterns were reproduced with moderately high accuracy: One had a high local gain (high variance of the excitatory and inhibitory threshold) and weak global coupling strength, while the other had a low local gain (low variance of the excitatory and inhibitory threshold) and strong global coupling strength. In investigating the neural mass dynamics generated from these two parameter sets, the former showed highly periodic and synchronized activation; the latter showed fewer synchronized and periodic activations. The temporal transition of the simulated microstates for the former parameters persisted for about 200 ms, and that for the latter parameters persisted for 150 ms. Both resulted in longer durations than the experimental data. Our results demonstrate that a bottom-up approach, which extends microscopic models of single-neuron dynamics based on empirical studies (Hodgkin and Huxley, 1952; Morris and Lecar, 1981) into a mesoscopic neural mass dynamics model (Larter et al., 1999) and integrates macroscopic structural connectivity, can effectively reveal both macroscopic fast and slow resting-state network dynamics that are observed in human neuroimaging measurements.

## Materials and Methods

**Figure 1:**
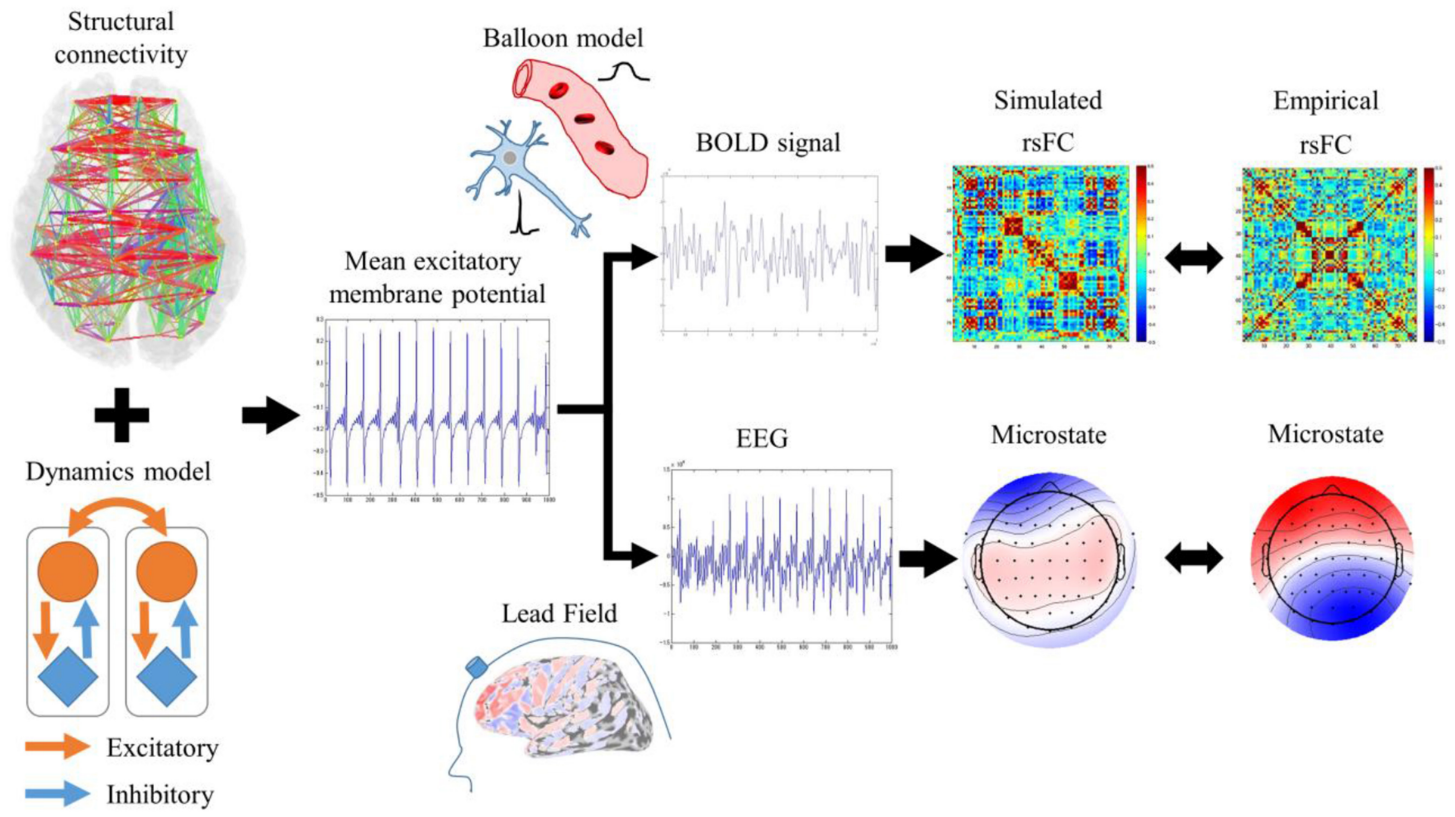
Evaluation procedures for rsFC and microstates. Mean excitatory membrane potentials obtained by Larter-Breakspear model integrating structural connectivity were converted into simulated rsFC and microstates by Balloon-Windkessel model and lead field and compared with empirical rsFC and microstates.

RSNs are characterized by an rsFC based on slow fluctuations observed by fMRI and microstates based on fast synchronization transitions observed by EEG. Simulated rsFCs and microstates, which were obtained by the Larter-Breakspear model that integrated the empirical structural connectivity, were compared with the empirical rsFCs and the microstates. Regarding slow fluctuation, the mean excitatory membrane potentials were converted into blood-oxygen-level dependent (BOLD) signals by the Balloon-Windkessel model. Next, after global fluctuations were regressed out of the BOLD signals, simulated rsFCs were obtained by calculating the cross-correlation coefficients among the BOLD signals. The empirical and simulated rsFCs were evaluated for their spatial pattern similarity. Regarding the fast synchronization transitions, a simulated EEG was obtained by multiplying the lead field and transformed into microstates by applying modified k-means clustering. We evaluated the empirical and simulated microstates for their spatial pattern similarity and non-stationary switching of microstates (Figure 1).

### Data Acquisition

#### Structural and Diffusion MRI Data

To obtain a structural connectivity matrix using a fiber-tracking algorithm, we measured the T1-weighted structural (TR: 2,300 ms, TE: 2.98 ms, Flip angle: 9°, TI: 900 ms, thickness: 1 mm, FOV: 256, matrix: 256 × 256, iso-voxel) and diffusion MRI data (gradient directions: 64, *b*-value: 1,000, thickness: 2 mm, iso-voxel) that were acquired on a 3T Trio (Siemens, Erlangen, Germany) from 13 participants (11 males and 2 females, aged 28.7 ± 8.47 years). All of the 13 participants gave informed written consent. All of the experiments in this study were conducted according to the Declaration of Helsinki and were approved by the Ethics Committee of the Advanced Telecommunications Research Institute International, Japan.

#### Resting-State Functional MRI Data

We recorded the resting-state brain activities for 10 min. The same 13 participants who took part in the dMRI experiment fixated on a cross, let their mind wander, and avoided focusing on any one thing. The resting-state functional imaging data (TR: 2,500 ms, TE: 30 ms, FOV: 212 mm, flip angle: 80°, matrix: 64 × 64, thickness: 3.2 mm, gap: 0.8 mm, 40 slices × 244 volumes) were acquired on a 3T Trio (Siemens, Erlangen, Germany).

#### Resting-State EEG Data

The resting-state EEGs were recorded for 5 min. The four participants who took part in the fMRI experiment fixated on the cross, let their mind wander, and avoided focusing on any one thing. All of the four participants gave informed written consent. Their EEGs were recorded with a whole-head 63-channel system (BrainAmp; Brain Products GmbH, Germany). The sampling frequency was 1 kHz. Electrooculogram (EOG) signals were simultaneously recorded and then stored in the EEG.

### Data Analysis

#### Empirical Structural Connectivity

We computed the experimental structural connectivity matrix in accordance with a previous work (Fukushima et al., 2015). Briefly, the seed and target ROIs used for fiber-tracking were obtained by FreeSurfer. The participants' motions were corrected by the FMRIB Software Library (FSL). Fractional anisotropy images were then calculated from the corrected images and used for registering the diffusion-space to the T1-space by a non-linear registration tool (FNIRT) in FSL. The local model of the fiber orientations was the fiber orientation distribution (FOD), reconstructed at each voxel by constrained spherical deconvolution (Tournier et al., 2007) with six-dimensional spherical harmonics for the response function. Based on the reconstructed FOD, fibers were probabilistically tracked by MRtrix. The fiber tracks were generated 105 times from each ROI. We calculated the structural connectivity strength as the number of fibers within each ROI pair *ft* divided by the total number of fibers generated from seed ROI *fs* with voxel size normalization: (*ft*/*vt*)/(*fs*/*vs*), where *vt* and *vs* are the number of voxels in the target and the seed ROI. Since the direction of the structural connectivity strength was not determined by a measurement principle, the structural connectivity matrix was symmetrized by assigning the higher strength to both directions. All parameters were determined as done in a previous work (Fukushima et al., 2015). The representative structural connectivity matrix was obtained by averaging the structural connectivity matrices of all participants with respect to the participants' common cortical ROI (Table S1).

#### Empirical Resting-State Functional Connectivity

The resting-state functional imaging was preprocessed with SPM8 software (Wellcome Trust Center for Neuroimaging, University College London, UK) in MATLAB (R2013a, Mathworks, USA) as follows. First, the raw functional images were corrected for slice-timing and realigned to the mean image of that sequence to compensate for the head motion. Second, the structural images were co-registered to the mean functional image and segmented into three tissue classes in the Montreal Neurological Institute (MNI) space. The functional images were then normalized and resampled in a 2 × 2 × 2 mm grid and smoothed by a Gaussian of 8 *mm* full-width at half-maximum.

We computed the functional connectivity matrix using parcellation defined by anatomical automatic labeling (AAL) for each participant. We extracted a representative time course in each region by averaging the time courses of the voxels therein. A band-pass filter (transmission range, 0.008–0.1 Hz) was applied to these sets of time courses prior to the following regression procedure. The filtered time courses were linearly regressed by the temporal fluctuations of the white matter, the cerebrospinal fluid, and the entire brain. Here, the fluctuation in each tissue class was determined from the average time course of the voxels within a mask created by the segmentation procedure of the T1 image. These extracted time courses were bandpass-filtered (transmission range, 0.008–0.1 Hz) before the linear regression, as was done for the regional time courses. All parameters were determined as done in a previous work (Yahata et al., 2016). Then a representative rsFC was obtained by calculating the cross-correlation coefficients among the BOLD signals for each participant and averaging the rsFC of each participant with respect to the common cortical ROI in all participants.

#### Microstates

The EEG data were preprocessed with a low-pass FIR filter with a cutoff frequency of 50 Hz, downsampled at 100 Hz, and passed through a high-pass FIR filter with a cutoff frequency of 0.5 Hz. After a common average reference, the EOG artifacts were removed by generating a multiple linear regression model to predict the eye-movement-related components in the EEG data using the EOG data. Cardiac artifacts and sensor noise were removed by ICA. All of the EEG data were converted into empirical microstates.

Since microstates are seen as building blocks of human information processing as noted in a previous work (Koenig et al., 2002), they were introduced as the criteria to compare the fast dynamics between empirical and simulated neuronal dynamics. First, the standard deviation of all EEG signals, called the global field power (GFP), was calculated as the criterion of the signal-to-noise ratio (SNR). Second, four microstates were identified by the basic N-microstate algorithm applied to the normalized EEG at the local maxima in the GFP curve. The optimal number of microstates was set to four based on a large-scale study on microstates (Koenig et al., 2002), and probabilistically discrete initial values for clustering were chosen based on the k-means++ algorithm (Arthur and Vassilvitskii, 2007). If the spatial correlation value between one microstate and another microstate was 0.9 or more, these two microstates were merged. Third, the transition of the microstates was calculated by a segmentation-smoothing algorithm. All parameters were determined as done in a previous work (Pascual-Marqui et al., 1995). That is, convergence criterion parameter ϵ = 10^−6^, window size parameter *b* = 3, and non-smoothness penalty parameter λ = 5. The occupation ratio is defined as the time allocated to a microstate divided by the total time.

### Computational Modeling

#### Larter-Breakspear Model

The Larter-Breakspear model is a phenomenological scheme that describes the electrophysiological neuronal dynamics in each region based on structural connectivity. This model consists of the mean membrane potential of the excitatory neurons (V) and the inhibitory neurons (Z), and the average number of open potassium ion channels (W). The mean firing rate for excitatory and inhibitory populations are described by *Q*_*V*_ and *Q*_*Z*_. The voltage-dependent fractions of open ion channels are described by *m*_*ion*_. These sigmoidal functions describe averaging over a population of ion channels and cell firing rates under Gaussian distribution. Excitatory interactions between region *i* and *j* are described by ${\langle Q_{V}\rangle }^{i}$. Simulations were performed using ode23, which automatically chooses the step size, maintains a specified accuracy, and solves ordinary differential equations in MATLAB. We repeated simulations for each parameter set 10 times with different initial values to reduce the influence of initial values. The simulation length was set to 10 min. We discarded the first 2 min to eliminate the influence of the initial values. All simulation parameters in Table 1 were determined based on a previous work (Roberts et al., 2019). In the parameter search, the balance of intra- and inter-regional excitatory synaptic connection strength was changed by the global coupling strength C, and the oscillation of the excitatory and inhibitory neural populations was changed by the variance of the excitatory and inhibitory threshold. It took about 2 days to complete 10 min of simulation for a particular parameter set and an initial value using our high performance computer server.

(1) $$\begin{array}{r} m_{ion}=0.5\left(1+tanh\left(\frac{V^{i}-T_{ion}}{\delta_{ion}}\;\right)\right), \end{array}$$

(2) $$\begin{array}{r} Q_{V}=0.5Q_{V_{max}}\left(1+tanh\left(\frac{V^{i}-V_{T}}{\delta_{V}}\right)\right), \end{array}$$

(3) $$\begin{array}{r} Q_{Z}=0.5Q_{Z_{max}}\left(1+tanh\left(\frac{Z^{i}-Z_{T}}{\delta_{Z}}\right)\right), \end{array}$$

(4) $$\begin{array}{l} \frac{dV^{i}}{dt}=-(g_{Ca}+\left(1-C\right)r_{NMDA}a_{ee}Q_{V}^{i}\\ \;+Cr_{NMDA}a_{ee}{\langle Q_{V}\rangle }^{i})m_{Ca}\left(V^{i}-V_{Ca}\right)\\ \;-g_{K}W\left(V^{i}-V_{K}\right)-g_{L}\left(V^{i}-V_{L}\right)\\ \;-\left(g_{Na}m_{Na}+\left(1-C\right)a_{ee}Q_{V}^{i}+Ca_{ee}{\langle Q_{V}\rangle }^{i}\right)\left(V^{i}-V_{Na}\right)\\ \;-a_{ie}ZQ_{Z}^{i}+a_{ne}I, \end{array}$$

(5) $$\begin{array}{r} \frac{dZ^{i}}{dt}=b\left(a_{ni}I+a_{ei}V^{i}Q_{V}^{i}\right), \end{array}$$

(6) $$\begin{array}{r} \frac{dW^{i}}{dt}=\phi \frac{m_{K}-W^{i}}{\tau_{K}} \end{array}$$

(7) $$\begin{array}{r} {\langle Q_{V}\rangle }^{i}=\frac{\sum_{j}u_{ij}Q_{V}^{j}}{\sum_{j}u_{ij}}. \end{array}$$

Here, *u*_*ij*_ is the structural connectivity strength between region *i* and region *j*.

**Table 1:** Parameter values for the Larter-Breakspear model.

**Parameter**	**Description**	**Value**
T_Ca_	Threshold value for Ca channels	−0.01
δ_Ca_	Variance of Ca channel threshold	0.15
g_Ca_	Conductance of population of Ca channels	1
V_Ca_	Ca Nernst potential	1
T_K_	Threshold value for K channels	0.0
δ_K_	Variance of K channel threshold	0.30
g_K_	Conductance of population of K channels	2.0
V_K_	K Nernst potential	−0.7
T_Na_	Threshold value for Na channels	0.3
δ_Na_	Variance of Na channel threshold	0.15
g_Na_	Conductance of population of Na channels	6.7
V_Na_	Na Nernst potential	0.53
V_L_	Nernst potential leak channels	−0.5
g_L_	Conductance of population of leak channels	0.5
V_T_	Threshold potential for excitatory neurons	0.0
Z_T_	Threshold potential for inhibitory neurons	0.0
δ_Z_	Variance of inhibitory threshold	Same value as δ_V_
Q_V_max__	Maximal firing rate for excitatory populations	1.0
_Q_Z_min_	Maximal firing rate for inhibitory populations	1.0
I	Subcortical input strength	0.30
a_ee_	Excitatory-to-excitatory synaptic strength	0.36
a_ei_	Excitatory-to-inhibitory synaptic strength	2
a_ie_	Inhibitory-to-excitatory synaptic strength	2
a_ne_	Non-specific-to-excitatory synaptic strength	1
a_ni_	Non-specific-to-inhibitory synaptic strength	0.4
b	Time constant scaling factor	0.1
φ	Temperature scaling factor	0.7
τ_K_	Time constant for K relaxation time	1
r_NMDA_	Ratio of NMDA to AMPA receptors	0.25
δ	Random modulation of subcortical input	0

#### Simulated Bold

To calculate the simulated rsFC, the mean membrane potential of the excitatory neurons was converted into simulated BOLD signals by the Balloon-Windkessel hemodynamic model. In this paper, neuronal activity was given by the absolute value of the time derivative of the mean excitatory membrane potential within each brain region. For the *i*th region, neuronal activity *z*_*i*_ increased vasodilatory signal *s*_*i*_, which is subject to autoregulatory feedback. Inflow *f*_*i*_ responds in proportion to this signal with concomitant changes in blood volume *v*_*i*_ and deoxyhemoglobin content *q*_*i*_. The following are the related equations:

(8) $$\begin{array}{r} \frac{ds_{i}}{dt}=z_{i}-\kappa_{i}s_{i}-\gamma_{i}\left(f_{i}-1\right), \end{array}$$

(9) $$\begin{array}{r} \frac{df_{i}}{dt}=s_{i}, \end{array}$$

(10) $$\begin{array}{r} \tau_{i}\frac{dv_{i}}{dt}=f_{i}-v_{i}^{1/\alpha }, \end{array}$$

(11) $$\begin{array}{r} \tau_{i}\frac{dq_{i}}{dt}=\frac{f_{i}\left(1-\left(1-\rho_{i}^{1/f_{i}}\right)\right)}{\rho_{i}}-\frac{v_{i}^{1/\alpha }q_{i}}{v_{i}}, \end{array}$$

where κ_*i*_ = 0.65 is the rate of signal decay, α = 0.32 is Grubb's exponent, τ_*i*_ = 0.98 is the hemodynamic transit time, and ρ = 0.34 is the resting oxygen extraction fraction. The BOLD signal is a static non-linear function of volume and deoxyhemoglobin comprised of a volume-weighted sum of the extra- and intra-vascular signals:

(12) $$\begin{array}{r} y_{i}=V_{0}\left(7\rho_{i}\left(1-q_{i}\right)+2\left(1-\frac{q_{i}}{v_{i}}\right)+\left(2\rho_{i}-0.2\right)\left(1-v_{i}\right)\right), \end{array}$$

where *V*_0_ = 0.02 is the resting-blood volume fraction. All of the simulation parameters were determined as done in a previous work (Friston et al., 2003).

#### Lead Field

The mean membrane potentials of the excitatory neurons were converted into simulated EEGs by the lead field that represents the linear relation between the sources on the cortical surface and the measurements on each EEG channel as a gain matrix. Polygon models of the cortical surfaces (20,004 vertex points) were constructed from T1 structural images of the same four participants who took part in the EEG experiment using FreeSurfer software (Dale et al., 1999). A single current dipole was assumed at each vertex point to be perpendicular to the cortical surface. The brain structures were approximated as a three-layer model by identifying three boundaries, i.e., for the cerebrospinal fluid (CSF), the skull, and the scalp, assuming that the conductivities were 0.33, 0.0042, and 0.33, respectively (Waberski et al., 1998). These surfaces obtained by FreeSurfer were slightly modified using gray/white/CSF segmentation by SPM8 (Welcome Department of Cognitive Neurology, UK) and morphological operations. All of the parameters were determined as done in a previous work (Aihara et al., 2012).

Singular value decomposition was applied to all points belonging to one ROI out of 20,004 points. The singular values were arranged in descending order and the lead field at 80% accumulation rate of singular values was averaged in one ROI. (That is, it has almost the same effect as averaging the lead field within an ROI). We calculated the low-dimensional lead field (63 channels × 78 ROIs) of each participant by averaging the components that contribute 80% for each ROI since the Later-Breakspear model has the same number of neurons in each ROI. This way, we removed the influence of heterogeneous ROI sizes in the empirical data. Then the low-dimensional lead field matrices of all subjects were averaged.

#### Simulated Microstates

To compare the empirical and simulated microstates, we converted the mean membrane potential of the excitatory neurons into simulated EEG signals by the lead field. First, the simulated EEG signals were calculated by multiplying the mean membrane potential of the excitatory neurons by the lead field. Second, a common average reference was applied for simulated EEG signals and the simulated EEG signals were bandpass-filtered between 10 and 15 Hz. Third, the bandpass-filtered signals were downsampled from 1,000 to 100 Hz. Finally, the simulated microstates were acquired by applying the basic N-microstate algorithm and the segmentation smoothing algorithm to the obtained signals.

#### Quantitative Evaluation of Empirical and Simulated Results

The spatial correlation between the empirical and simulated rsFCs was obtained by averaging the cross-correlation coefficients of the lower triangular components between the empirical and simulated rsFCs for each parameter set.

We evaluated the empirical and simulated microstates in terms of spatial similarity, occupation ratio, mean transition time, and global explained variance (GEV). GEV represents the goodness of fit between the microstates and the normalized EEG weighted by GFP (Khanna et al., 2014). We obtained the spatial correlation between the empirical and simulated microstates by the following procedures. For the 24 combinations between the empirical and simulated microstates, we calculated the average cross-correlation coefficients of each combination and chose the one that maximized it. In the simulation, two microstates that showed similar spatial patterns (similarity over 0.9) were merged. This happened for several parameter combinations. If the number of simulated microstates was >4, the cross-correlation coefficients were set to 0 for the missing simulated microstates. The occupation ratio was obtained by dividing the time allocated to each microstate by the total simulation time. The mean transition time was obtained by averaging required time to transition from one microstate to another.

#### Phase-Locking Matrix

The phase-locking matrix quantitatively visualizes the inter-regional interdependence of the non-linear simulated neuronal dynamics. First, the 10–15 Hz bandpass filter was applied to the mean membrane potentials. Second, these signals were Hilbert-transformed, extracting the phase θ. Finally, we calculated the phase-locking values (PLVs) between regions p and q by the following formula:

(13) $$\begin{array}{r} \text{PLVs}=\frac{\left|\sum_{t=1}^{N_{T}}e^{i\left(\theta_{q}^{t}-\theta_{p}^{t}\right)}\right|}{N_{T}}, \end{array}$$

where *N*_*T*_ represents the sampling number.

## Results

### Spatial Pattern Similarity of rsFC and Microstates

**Figure 2:**
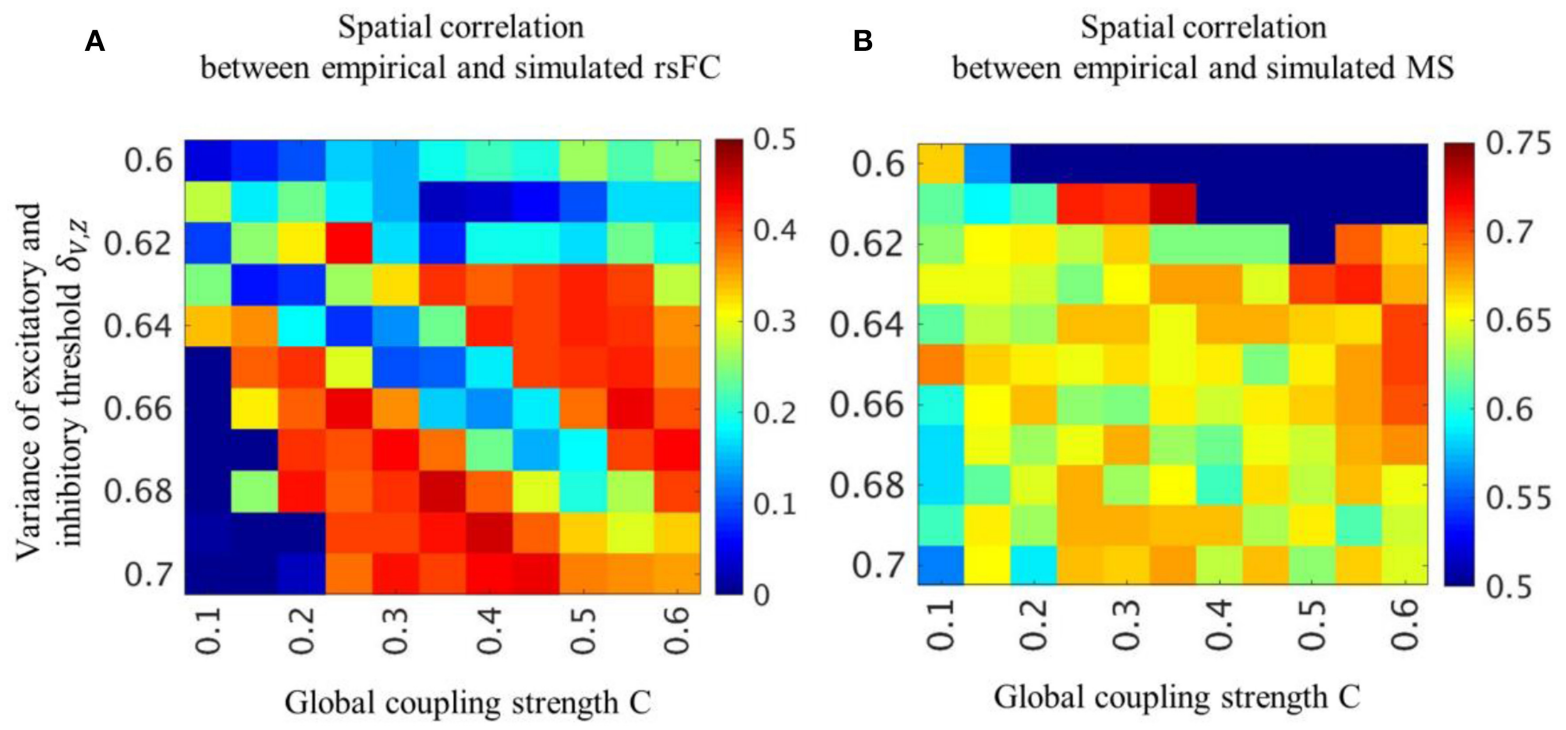
Spatial pattern similarity of rsFC and microstates for each parameter combination. Averaged cross-correlation coefficients between empirical and simulated rsFC **(A)** and microstates **(B)** are obtained by varying global coupling strength *C* and variance of threshold **δ**_*V, Z*_. Color bars indicate strength of cross-correlation coefficients.

In the two parameter groups of strong global coupling strength and small variance of threshold or weak global coupling strength and large variance of threshold, the simulated rsFC and microstates indicated a high spatial similarity to the empirical rsFCs and microstates (Figure 2). We selected the optimal parameter groups by considering both the spatial similarities of rsFC, which were moderately high over a broad range, and the spatial similarities of the microstates, which were high in a narrow range. The spatial similarities in the optimal parameter groups obtained with structural connectivity based on diffusion MRI were significantly higher than those obtained with shuffled structural connectivity (Figure S1).

The spatial pattern similarity between empirical and simulated microstates in the (C, δ_V, Z_) = (0.35, 0.61) condition is high but the occupation ratio and the mean transition time are greatly different from empirical microstates (Figure 2B, Figure S2). The occupation ratio and the mean transition time in the (C, δ_V, Z_) = (0.30, 0.61) and (0.25, 0.61) are almost the same as in the (C, δ_V, Z_) = (0.35, 0.61) condition.

### Comparison of Spatiotemporal Patterns Between Empirical and Simulated Microstates

Empirical and simulated microstates were obtained by applying the basic *N*-microstate algorithm and the segmentation smoothing algorithm for EEG time-series. Roughly, one microstate probabilistically transitions to another microstate when GFP is at a local minimum (Figure S3).

**Figure 3:**
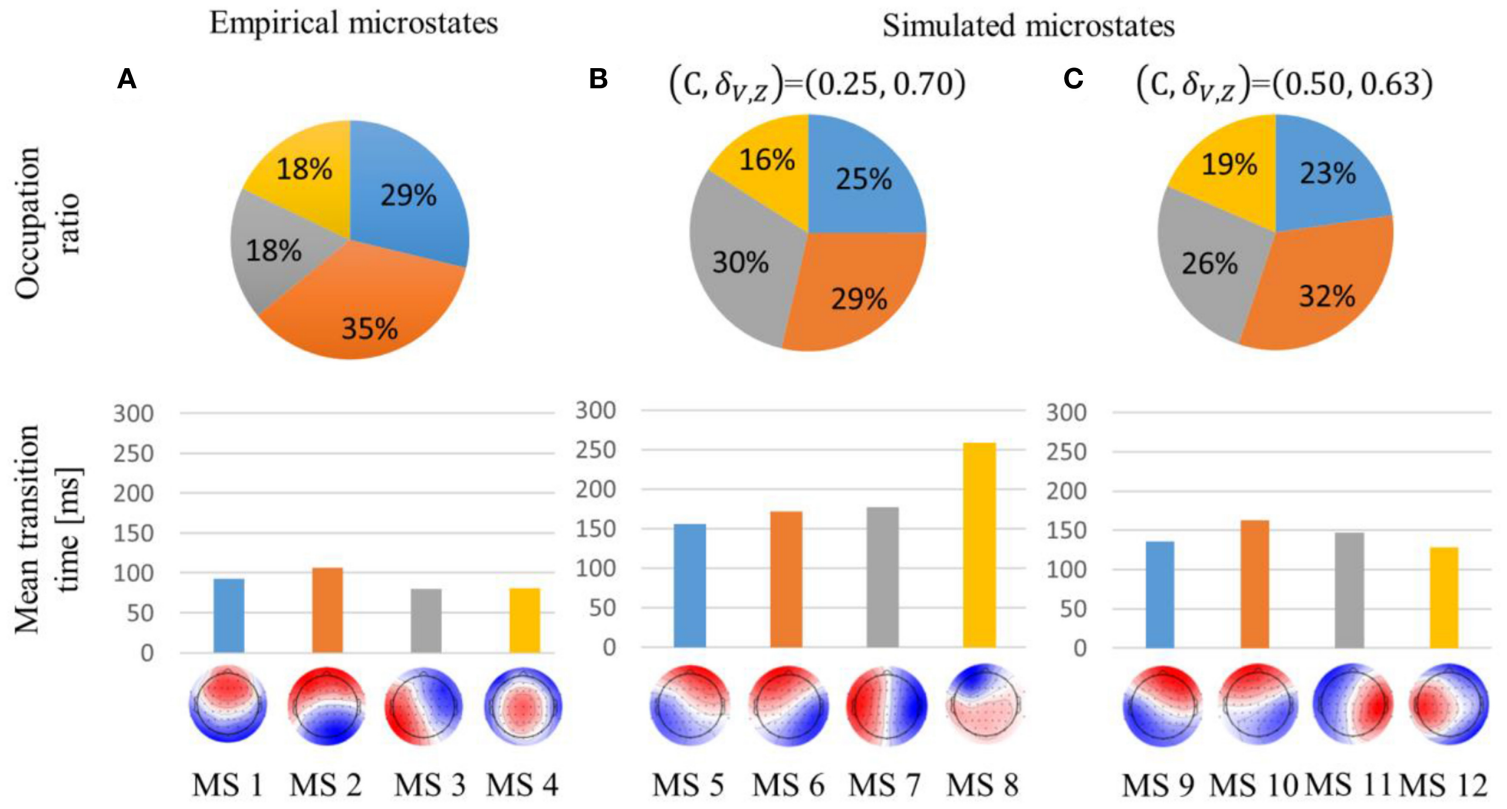
Occupation ratio and mean transition time of empirical **(A)** and simulated **(B,C)** microstates. global explained variance (GEV) was 59, 66, and 69% for the empirical, simulated weak global coupling strength, and simulated strong global coupling strength microstates, respectively. Each simulated microstate is indicated with the same color as the empirical microstate with which it is most highly correlated. Interchanging red and blue would correspond to the identical pattern.

Concerning the empirical microstates, MS2 accounted for the highest proportion, and their mean transition times were about 100 ms, as in the previous research (Figure 3A).

In the (C, δ_V, Z_) = (0.25, 0.70) condition, the simulated microstates were sustained about twice as long compared with the empirical microstates. In contrast to the empirical microstates, MS6 and MS7 accounted for a higher proportion than MS5. Furthermore, MS8's temporal transition was also intermediate between MS2 and MS4 because MS8 accounted for the lowest proportion, even though it was sustained for the longest time (Figure 3B).

In the (C, δ_V, Z_) = (0.50, 0.63) condition, the simulated microstates were sustained about 1.5 times longer than the empirical microstates. Unlike the (*C*, δ_V, Z_) = (0.25, 0.70) condition, the occupation ratio of MS10 was about 5% higher than that of MS11. Moreover, the spatial pattern of MS12 was biased to the left hemisphere (Figure 3C).

### Comparison of Neuronal Dynamics and Phase-Locking

**Figure 4:**
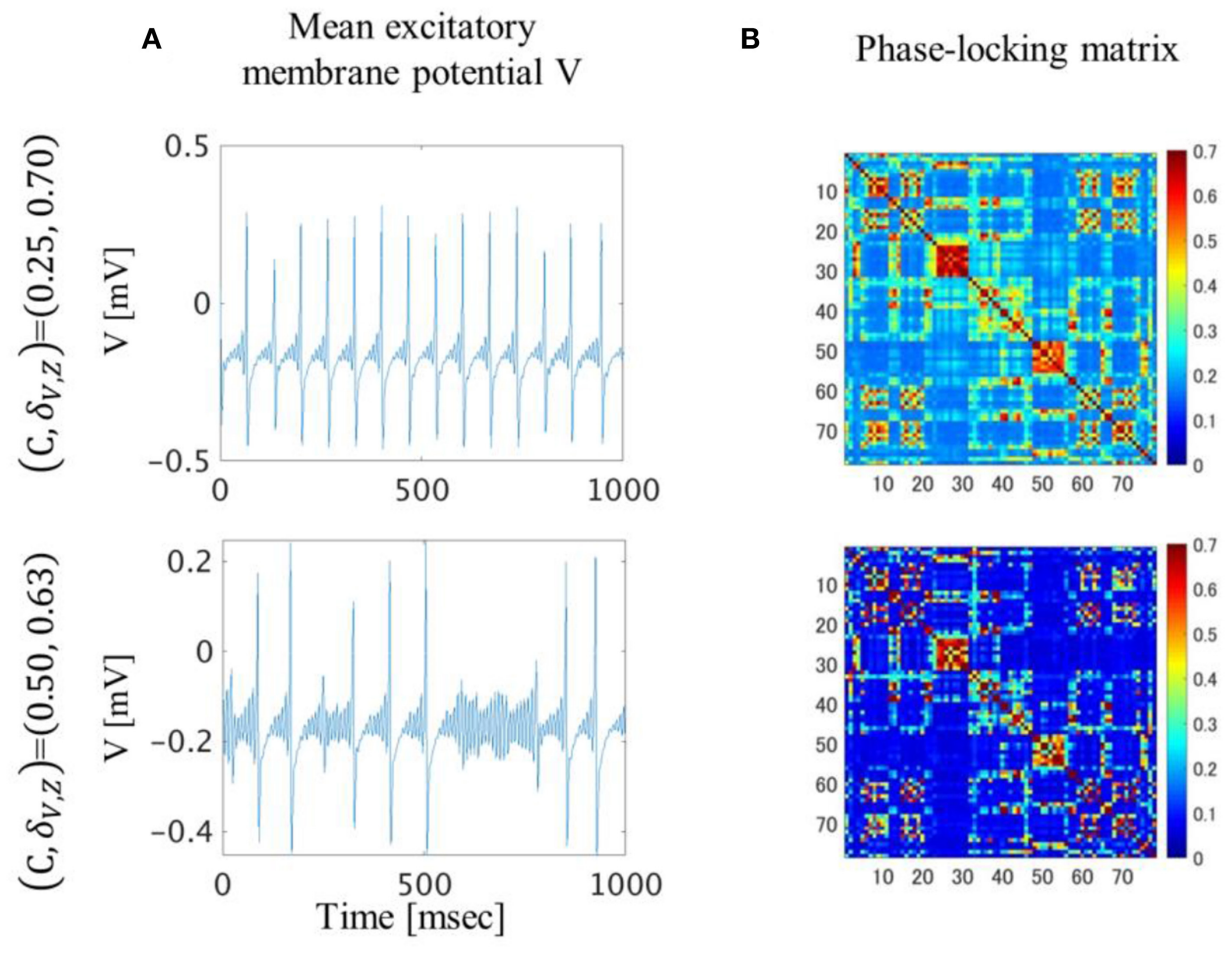
Excitatory mean membrane potentials for one second **(A)** and averaged phase-locking matrix **(B)**. In the (C, δ_V, Z_)**=**(0.25, 0.70) condition, excitatory mean membrane potentials fluctuated in a regular manner and were synchronized across the whole brain region. In the (C, δ_V, Z_)**=**(0.50, 0.63) condition, excitatory mean membrane potentials fluctuated irregularly and were synchronized between brain regions with strong structural connectivity.

In the (C, δ_V, Z_) = (0.25, 0.70) condition, the excitatory mean membrane potentials were synchronized across the whole brain region with weak global coupling strength because the self-recurrent excitation was higher than the low variance of the threshold (Figures 4A,B top). In the (C, δ_V, Z_) = (0.50, 0.63) condition, the excitatory mean membrane potentials were synchronized between the brain regions with strong structural connectivity and were not synchronized between the brain regions with weak or no structural connectivity (Figure 4B bottom, Figure S1A). Strong global coupling strength and structural connectivity are required for excitatory mean membrane potentials to exceed the excitatory threshold due to weak self-recurrenct excitation. Due to the synchronized excitatory mean membrane potentials across the whole brain region, the mean transition time in the (C, δ_V, Z_) = (0.25, 0.70) condition is 1.3 times longer than the mean transition time in the (C, δ_V, Z_) = (0.50, 0.63) condition (Figure 3).

## Discussion

### Summary

In this study, we investigated whether there is a model that simultaneously explains two experimentally observed phenomena in the resting state: slow fluctuation manifested by resting-state functional connectivity (rsFC) and fast transient dynamics manifested by EEG microstates. We simulated a neural mass model using the Larter-Breakspear model constrained by the structural connectivity and optimized the model parameters [the global coupling parameter and the local gain parameter (variance of excitatory and inhibitory threshold)] by fitting the simulated rsFC to the experimental rsFC and the simulated microstates to the experimental microstates. As a result, we obtained three key findings: first, the parameter sets with high fitting performance to rsFC overlapped with those with high fitting performance to the microstate; second, two distinct parameter sets were identified within the overlapped parameter region; third, the overlapped parameter sets are much narrower than the parameter sets obtained by fitting only rsFC. In other words, based on these three findings, both fast synchronization transitions and slow BOLD fluctuation changed based on structural connectivity in the overlapped parameter regions. Empirical microstates were similar to simulated microstates in these regions. Thus, fast synchronization transitions correlated with slow BOLD fluctuation based on structural connectivity yielded microstates. These results suggest that adding the microstates as fitting criteria is important for significantly reducing the uncertainty of good model parameters. The maximal cross-correlation coefficient of the rsFC was about 0.45, which is almost the same value as that in previous research (Honey et al., 2009). The maximal cross-correlation coefficient of the microstates was about 0.7, and the mean transition times of the simulated microstates were about 1.5 or 2.0 times longer than those of the empirical microstates.

### Comparison With Previous Research

Since the structural connectivity in this study had stronger interhemispheric connectivity (Figure S1A) than in the previous research, the cross-correlation coefficients of rsFC were about 0.05 higher than in the previous research (Honey et al., 2009; Deco et al., 2013).

In the (C, δ_V, Z_) = (0.50, 0.63) condition, the dynamics of the excitatory mean membrane potentials resembled the dynamics of previous research in terms of irregular firing (Roberts et al., 2019). The simulated microstates using shuffled structural connectivity did not reproduce either a spatial pattern or a temporal transition (Figure S4). In the (C, δ_V, Z_) = (0.25, 0.70) condition, the dynamics of the excitatory mean membrane potentials resembled the dynamics of the previous research in terms of regular firing (Honey et al., 2009). Simulated microstates using shuffled structural connectivity reproduced a spatial pattern but not a temporal transition (Figures S4B, S5). Furthermore, the transition probability matrix of empirical microstates is more similar to the simulated one in the (C, δ_V, Z_) = (0.50, 0.63) condition than in the (C, δ_V, Z_) = (0.25, 0.70) condition (Figure S6). Therefore, the dynamics of the excitatory mean membrane potentials in the (C, δ_V, Z_) = (0.50, 0.63) condition resembled the empirical resting-state activity of the human brain. The dynamics of the excitatory mean membrane potentials in the (C, δ_V, Z_) = (0.25, 0.70) condition were comparatively unconstrained by the structural connectivity (Figure 4B, top). Although these were bandpass-filtered dynamics, perhaps they are related to the phenomena in which the activity spreads to most of the cortex when the cortex is stimulated with transcranial magnetic stimulation (TMS) during sleep (Alkire et al., 2008).

### Simulated Microstates

The MS7 and MS11 in each simulated case have a larger occupation ratio than the MS3 in the experiment (Figure 3) because the structural connectivity strength between the occipital regions is weak (Figure S1A). We assume that the interhemispheric structural connectivity was important for reproducing MS4, considering that MS4 has an equal distribution for the distant interhemispheric positions. Thus, the constraints based on structural connectivity are more important than the signal noise and the conduction delay proportional to the inter-regional distance. Signal noise probably affects temporal transition and the conduction delay proportional to the inter-regional distance probably results in ROIs close to each other being synchronized.

### Clinical Application for Cognitive Neuroscience

The development of biomarkers for psychiatric disorders using microstates and rsFC has been investigated (Yamada et al., 2017; D'Croz-Baron et al., 2019). One example is autism spectrum disorder (ASD). ASD patient's rsFC and microstates have different spatial and temporal characteristics compared to healthy individuals. Our results could not reproduce enough spatiotemporal characteristics of rsFC and microstates to compare ASD patients and healthy individuals. However, our results will give hints to solve these problems and enable making hypotheses about the dependence of slow and fast resting-state brain activity on neuronal network parameters.

### Effects of Averaging Lead Field

In the Larter-Breakspear model, the numbers of excitatory, and inhibitory neurons are the same in each ROI, and only the structural connectivity differs between the ROIs. For this reason, the high-dimensional lead field was averaged in each ROI to remove the area's influence in each ROI. If the ROIs were divided into smaller sections and the lead field was not averaged for each ROI, the simulated microstates might differ. We note that the contribution rate of the singular value decomposition did not affect the simulated microstates because the cross-correlation coefficient was 0.9995 between the lead fields by singular value decomposition and the mean value.

### Limitation of Current Simulation

The occupation ratio of the empirical and simulated microstates differed and the simulated microstate did not reproduce the specific spatial patterns of the empirical microstates. The resting-state alpha waves in the cerebral cortex are empirically affected by the thalamus (Sherman, 2016), and slow fluctuation is affected by the serotonin receptor density (Deco et al., 2018; Shine et al., 2019). However, the Larter-Breakspear model in this study does not include thalamic dynamics, conduction delay proportional to inter-regional distance, signal noise, or serotonin receptor density. Therefore, incorporating these effects in this model might more fully reveal how well the simulated rsFC and microstates reflect the empirical rsFC and microstates in realistic conditions.

### Numbers of Participants

Since empirical EEG and rsFC were obtained by two studies aiming at different purposes, the number of participants of EEG and rsFC differ. Empirical rsFC was obtained from 13 participants, sufficient for statistical purposes. Empirical EEG was obtained from only four participants. However, the four microstates identified from this empirical EEG are similar to the four microstates identified by other studies (Michel and Koenig, 2018b). We presume the four microstates will be similar to the microstates identified from 13 participants.

## Data Availability Statement

Relevant data and all codes used for the analyses are available from the authors on request.

## Ethics Statement

The studies involving human participants were reviewed and approved by the Ethics Committee of the Advanced Telecommunication Research Institute International, Japan. The patients/participants provided their written informed consent to participate in this study.

## Author Contributions

HE and OY: conceptualization, empirical data analysis, simulation programs, and simulation data analysis. OY: funding acquisition. NH: empirical data acquisition. HE, NH, and OY: writing the paper.

### Conflict of Interest

The authors declare that the research was conducted in the absence of any commercial or financial relationships that could be construed as a potential conflict of interest.
