# Supplementary material for: Evaluation of Resting Spatio-Temporal Dynamics of a Neural Mass Model Using Resting fMRI Connectivity and EEG Microstates
Source: Front Comput Neurosci. 2020 Jan 17;13:91. doi: 10.3389/fncom.2019.00091 (PMC6978716; doi:10.3389/fncom.2019.00091)
Supplement: Supplementary file 1 [file Table_1.docx]

**Supplementary material**

**Structural connectivity and shuffled structural connectivity matrices**


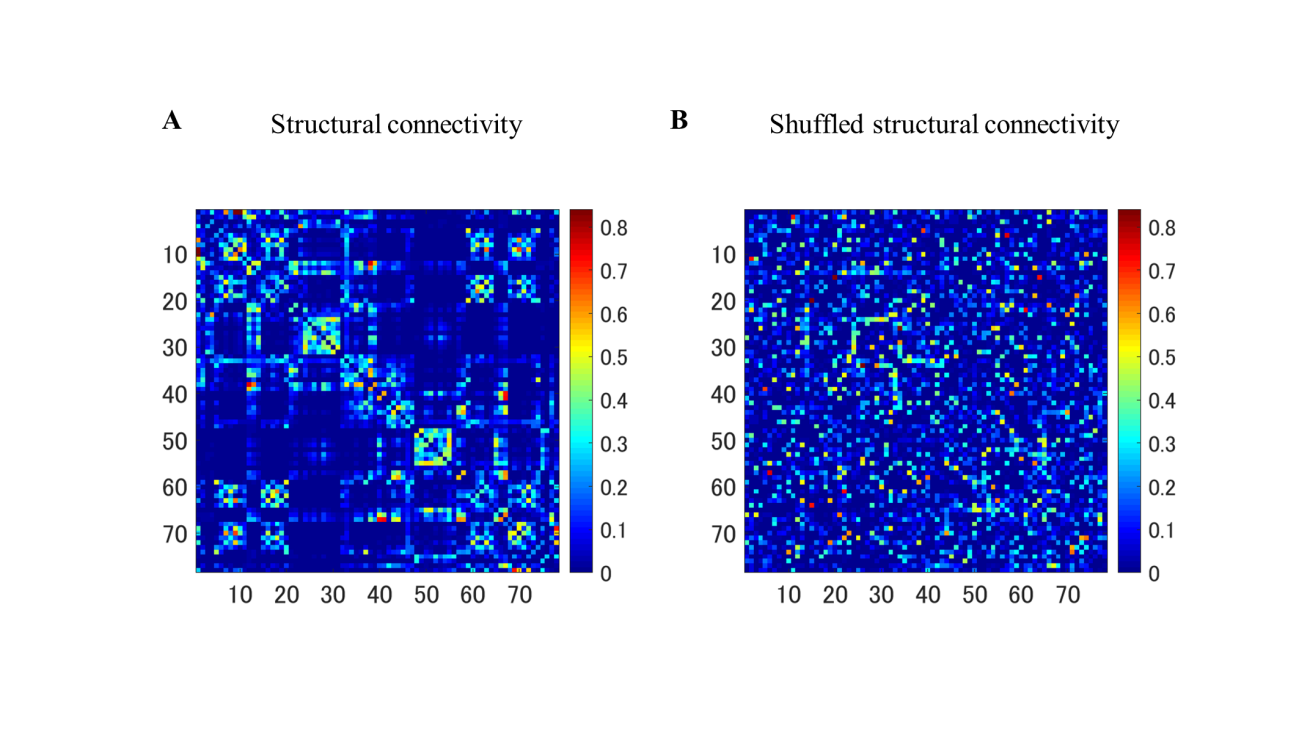


Figure S1. Structural connectivity matrix and shuffled structural connectivity matrix. Structural connectivity matrix obtained by averaging across 12 human participants, and shuffled structural connectivity matrix calculated by randomio_und_connected function of Brain Connectivity Toolbox while preserving distribution of degree of structural connectivity matrix.

Table S1. ROIs corresponding to region number in left hemisphere (ROI in right hemisphere arranged opposite to left hemisphere). As the Larter-Breakspear model is a cortical model, non-cortical regions were excluded from AAL. Furthermore, only cortical regions that were common to all subjects were chosen since our study focuses on common structure across all subjects.

| Region number | ROI |
| --- | --- |
| 1 | Olfactory |
| 2 | Cingulum Mid |
| 3 | ParaHippocampal |
| 4 | Temporal Pole Mid |
| 5 | Precentral |
| 6 | Frontal Sup |
| 7 | Frontal Sup Orb |
| 8 | Frontal Sup Medial |
| 9 | Frontal Med Orb |
| 10 | Rectus |
| 11 | Cingulum Ant |
| 12 | Cingulum Post |
| 13 | Precuneus |
| 14 | Temporal Mid |
| 15 | Frontal Mid |
| 16 | Frontal Mid Orb |
| 17 | Frontal Inf Oper |
| 18 | Frontal Inf Tri |
| 19 | Frontal Inf Orb |
| 20 | Supp Motor Area |
| 21 | Parietal Inf |
| 22 | Angular |
| 23 | Temporal Pole Sup |
| 24 | Temporal Inf |
| 25 | Calcarine |
| 26 | Cuneus |
| 27 | Lingual |
| 28 | Occipital Sup |
| 29 | Occipital Mid |
| 30 | Occipital Inf |
| 31 | Fusiform |
| 32 | Rolandic Oper |
| 33 | Insula |
| 34 | Postcentral |
| 35 | Parietal Sup |
| 36 | SupraMarginal |
| 37 | Paracentral Lobule |
| 38 | Heschl |
| 39 | Temporal Sup |

**Spatiotemporal patterns of simulated microstates in** $\left( \mathbf{C,}\boldsymbol{\delta}_{\mathbf{V, Z}} \right)\mathbf{=}\left( \mathbf{0.35, 0.61} \right)$ **condition**


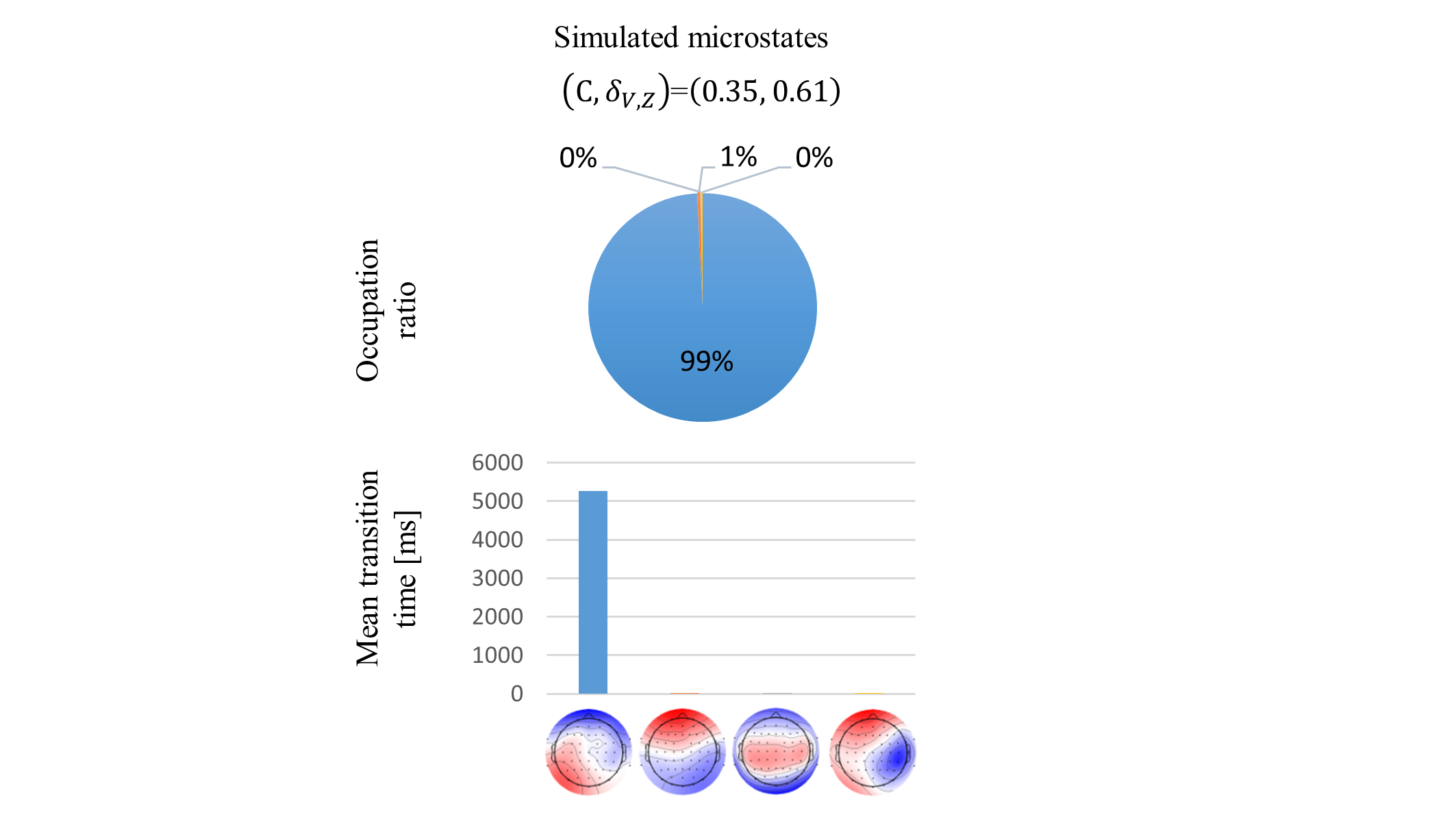


Figure S2. Occupation ratio and mean transition time of simulated microstates in $\left( \mathbf{C,}\boldsymbol{\delta}_{\mathbf{V, Z}} \right)\mathbf{=}\left( \mathbf{0.35, 0.61} \right)$ condition. Only 1 microstate accounts for simulated EEG and rarely transit to other microstates.

**Empirical and simulated EEG, GFP and microstate segmentation**


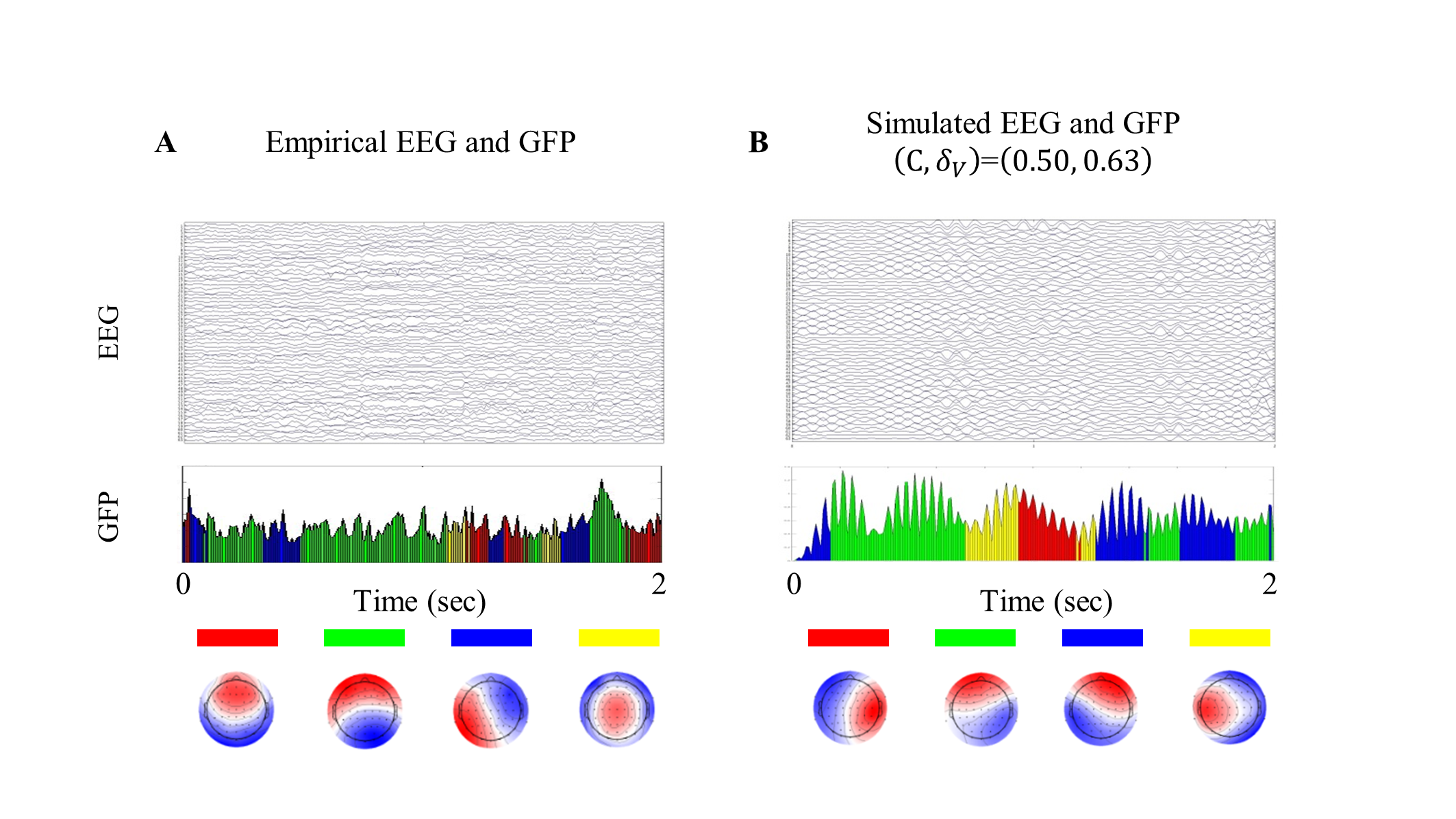


Figure S3. EEG, GFP and microstates segmentation in experiment (A) and in the simulated $\left( \mathbf{C,}\boldsymbol{\delta}_{\mathbf{V, Z}} \right)\mathbf{=}\left( \mathbf{0.50, 0.63} \right)$ condition (B). Each color corresponds to each microstate.

**Spatial pattern similarity of rsFC and microstates for shuffled structural connectivity**


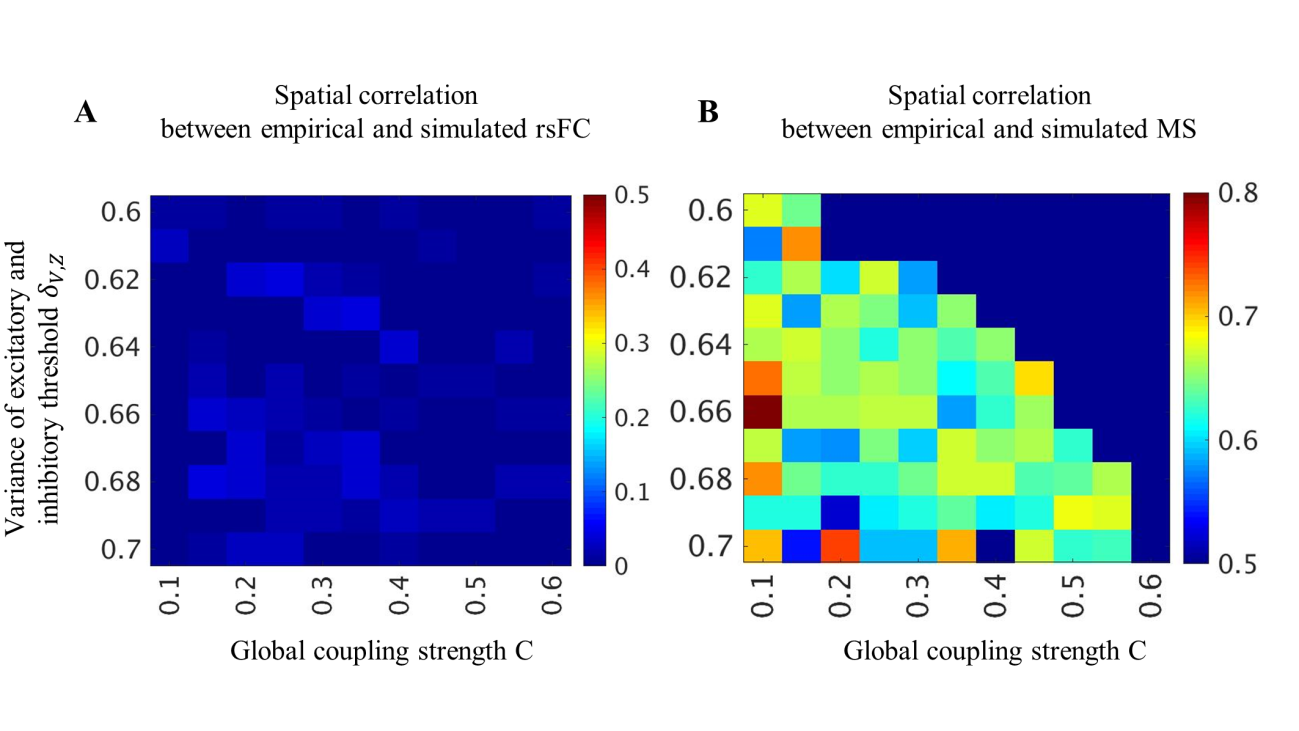


Figure S4. Cross-correlation coefficients between empirical and simulated spatial patterns (left rsFC, right microstates) based on shuffled structural connectivity matrix for combination of each parameter $\mathbf{(}\boldsymbol{C,}\boldsymbol{\delta}_{\boldsymbol{V, Z}}\mathbf{)}$. Simulation performed ten times for each parameter. rsFC’s spatial similarity was very low. Microstates’ spatial similarity was subequal as with non-shuffled structural connectivity matrix because simulated EEG was strongly constrained by lead field.


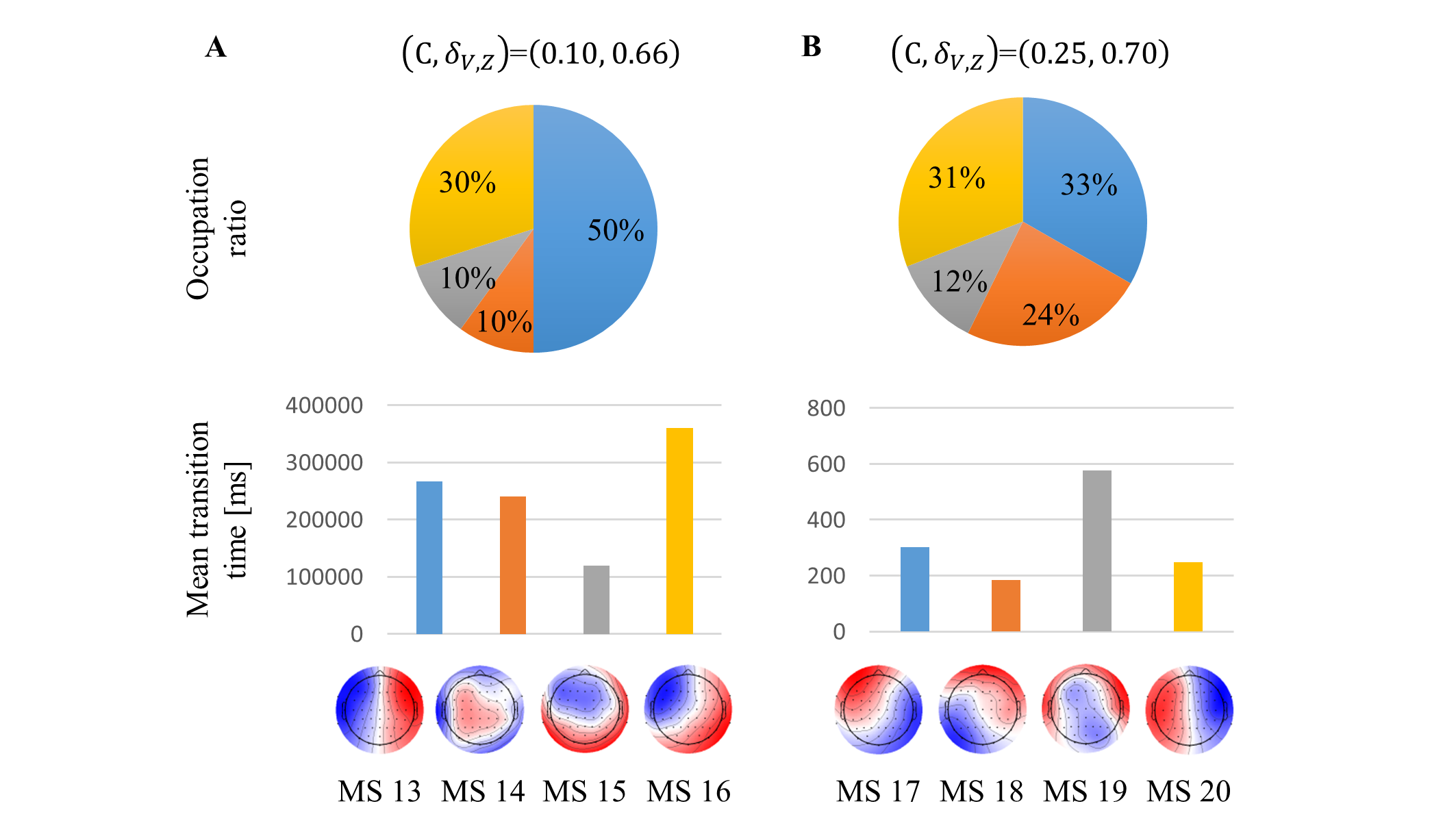


Figure S5. Occupation ratio and mean transition time in both conditions $\left( \boldsymbol{C,}\boldsymbol{\delta}_{\boldsymbol{V, Z}} \right)\mathbf{=}\left( \mathbf{0.10, 0.66} \right)$ and $\left( \mathbf{0.25, 0.70} \right)\mathbf{.}$ Structural connectivity matrix was shuffled for each trial. Microstates were simulated 100 times. In both conditions, potentially reversed microstates along longitudinal fissures of cerebrum like MS 13 and MS 20 accounted for a high percentage as opposed to interhemispheric equally potential microstate that accounted for a high percentage in empirical microstates. Mean transition time in condition $\left( \boldsymbol{C,}\boldsymbol{\delta}_{\boldsymbol{V, Z}} \right)\mathbf{=}\left( \mathbf{0.10, 0.66} \right)$ was much longer than mean transition time of empirical microstates.

**Transition probabilities of empirical and simulated microstates**


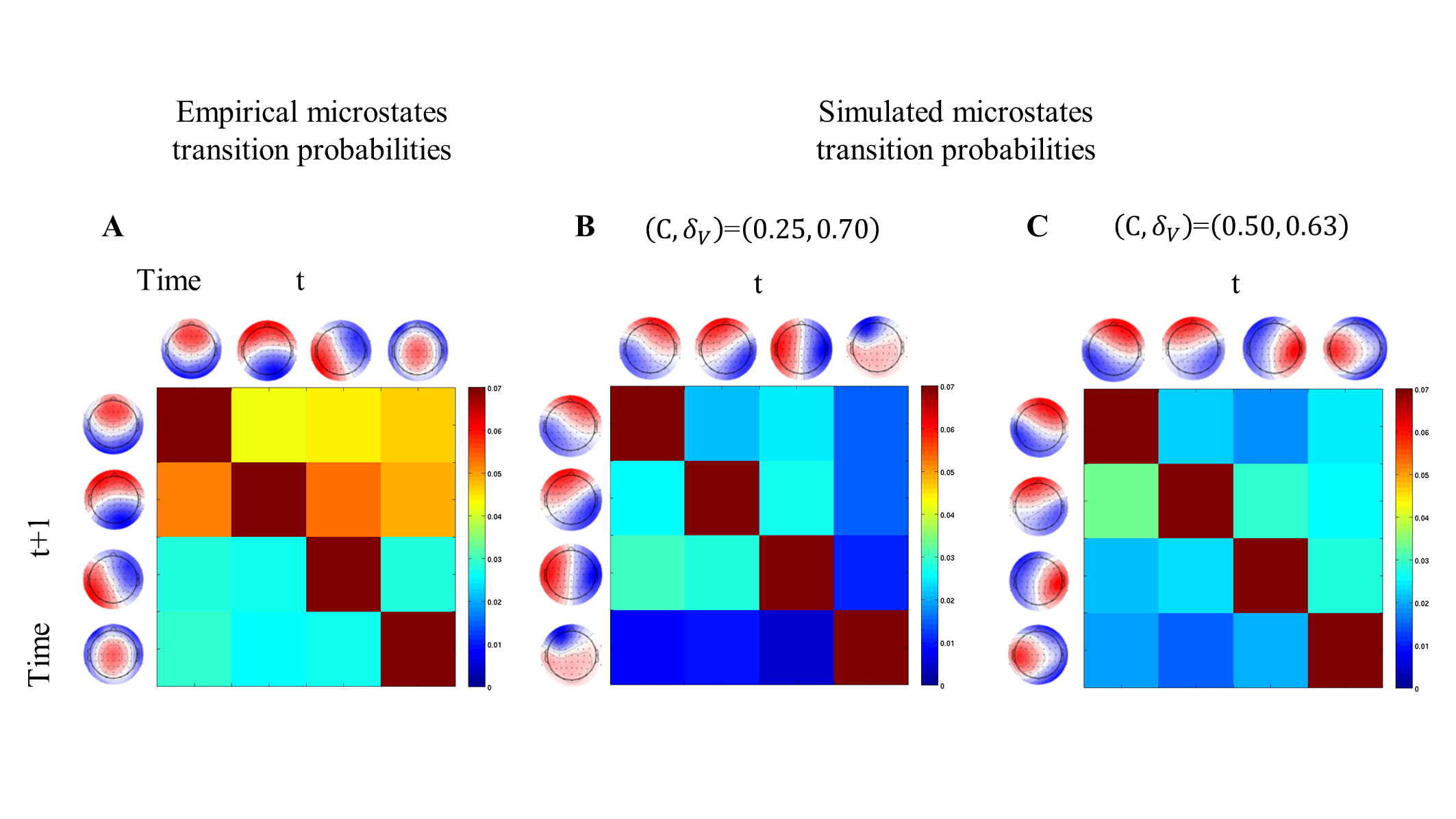


Figure S6. Transition probability matrices of empirical microstates (A), simulated microstates in the $\left( \boldsymbol{C,}\boldsymbol{\delta}_{\boldsymbol{V, Z}} \right)\mathbf{=}\left( \mathbf{0.25, 0.70} \right)$ condition (B), and simulated microstates in the $\left( \boldsymbol{C,}\boldsymbol{\delta}_{\boldsymbol{V, Z}} \right)\mathbf{=}\left( \mathbf{0.50, 0.63} \right)$ condition (C) per 10 msec. Transition probability matrices represent the probability that a microstate at time t will transit to another microstate at time t + 1.
